# Quantification of the type 2 diabetes risk in women with gestational diabetes: a systematic review and meta-analysis of 95,750 women

**DOI:** 10.1007/s00125-016-3927-2

**Published:** 2016-04-13

**Authors:** Girish Rayanagoudar, Amal A. Hashi, Javier Zamora, Khalid S. Khan, Graham A. Hitman, Shakila Thangaratinam

**Affiliations:** Women’s Health Research Unit, Blizard Institute, Barts and The London School of Medicine and Dentistry, Queen Mary University of London, 4 Newark Street, London, E1 2AB UK; Clinical Biostatistics Unit, Hospital Ramon y Cajal (IRYCIS), Madrid, Spain; CIBER Epidemiologia y Salud Publica (CIBERESP), Madrid, Spain; Multidisciplinary Evidence Synthesis Hub (mEsh), Blizard Institute, Barts and The London School of Medicine and Dentistry, Queen Mary University of London, London, UK

**Keywords:** Gestational diabetes, Meta-analysis, Postpartum, Predictors, Pregnancy, Risk factors, Systematic review, Type 2 diabetes

## Abstract

**Aims/hypothesis:**

Women with gestational diabetes mellitus (GDM) are at risk of developing type 2 diabetes, but individualised risk estimates are unknown. We conducted a meta-analysis to quantify the risk of progression to type 2 diabetes for women with GDM.

**Methods:**

We systematically searched the major electronic databases with no language restrictions. Two reviewers independently extracted 2 × 2 tables for dichotomous data and the means plus SEs for continuous data. Risk ratios were calculated and pooled using a random effects model.

**Results:**

There were 39 relevant studies (including 95,750 women) BMI (RR 1.95 [95% CI 1.60, 2.31]), family history of diabetes (RR 1.70 [95% CI 1.47, 1.97]), non-white ethnicity (RR 1.49 [95% CI 1.14, 1.94]) and advanced maternal age (RR 1.20 [95% CI 1.09, 1.34]) were associated with future risk of type 2 diabetes. There was an increase in risk with early diagnosis of GDM (RR 2.13 [95% CI 1.52, 3.56]), raised fasting glucose (RR 3.57 [95% CI 2.98, 4.04]), increased HbA_1c_ (RR 2.56 [95% CI 2.00, 3.17]) and use of insulin (RR 3.66 [95% CI 2.78, 4.82]). Multiparity (RR 1.23 [95% CI 1.01, 1.50]), hypertensive disorders in pregnancy (RR 1.38 [95% CI 1.32, 1.45]) and preterm delivery (RR 1.81 [95% CI 1.35, 2.43]) were associated with future diabetes. Gestational weight gain, macrosomia in the offspring or breastfeeding did not increase the risk.

**Conclusions/interpretation:**

Personalised risk of progression to type 2 diabetes should be communicated to mothers with GDM.

***Systematic review registration*:**

www.crd.york.ac.uk/PROSPERO CRD42014013597

**Electronic supplementary material:**

The online version of this article (doi:10.1007/s00125-016-3927-2) contains peer-reviewed but unedited supplementary material, which is available to authorised users.

## Introduction

Gestational diabetes mellitus (GDM), defined as glucose intolerance that is first diagnosed in pregnancy, is on the increase worldwide [1]. Up to half of all women with this condition progress to develop type 2 diabetes in later life, with the highest occurrence rate in the first 5 years after pregnancy [2]. Increasing numbers of women are presenting with previously undiagnosed diabetes and related complications, leading to substantial healthcare costs [3].

Current guidelines recommend following up women with GDM to identify type 2 diabetes at an early stage [4]. Ensuring compliance with this strategy is a significant global problem [5–7]. Despite evidence that the future risk of type 2 diabetes can be reduced by diet and lifestyle interventions and treatment with drugs such as metformin, [8, 9], less than a fifth of mothers with GDM undergo postpartum glucose screening [10]. Personalised risk communication with quantitative estimates can increase the number of individuals that make informed choices in screening programmes [11]. However, few prediction models for type 2 diabetes include GDM, and none of them account for pregnancy-specific characteristics [12].

Gestation-specific factors such as glycaemic status in pregnancy, gestational weight gain and obstetric complications are known to influence the future risk of diabetes [13–17]. Health-care professionals involved in the management of women with GDM do not provide individual risk estimates because of the lack of data; this greatly hinders counselling. Existing reviews have not quantified the risk of progression to type 2 diabetes [16, 18], and primary studies provide imprecise estimates because of their relatively small sample size [19–21]. We therefore undertook a systematic review to assess the strength of association of various maternal and pregnancy-related factors with GDM with the future risk of type 2 diabetes.

## Methods

We conducted a systematic review following a prospective protocol in line with current recommendations, and complied with the Preferred Reporting Items for Systematic Reviews and Meta-Analyses (PRISMA) guidelines for reporting (see electronic supplementary material [ESM] PRISMA checklist) [22]. Ethical approval was not required.

### Data sources and searches

We searched the MEDLINE and EMBASE databases (from inception until July 2015) for studies that assessed the risk factors in women with GDM and progression to type 2 diabetes. We used keywords, Medical Subject Headings and word variants for GDM such as ‘diabetes, gestational’, ‘GDM’, and ‘pregnancy induced diabetes’, and combined these with terms for type 2 diabetes such as ‘diabetes mellitus, type 2’, ‘NIDDM’ (i.e. non-insulin-dependent diabetes mellitus), ‘adult-onset diabetes mellitus’ and ‘ketosis-resistant diabetes’ (See ESM Search strategy). We searched the reference lists of identified papers for other relevant studies. Authors of eligible studies were contacted for further details if necessary. We did not apply any language restrictions.

### Study selection

Two independent reviewers (GR and AAH) selected the studies. First, we reviewed the abstracts for potentially relevant studies. Second, we obtained full copies of all possibly eligible papers for detailed evaluation. We included studies on women with GDM that assessed at least one of the following factors: maternal characteristics such as age, BMI, ethnicity, parity and family history of type 2 diabetes; factors specific to pregnancy such as gestational age at GDM diagnosis, antenatal OGTT, HbA_1c_, insulin use in pregnancy, gestational weight gain, pregnancy-induced hypertension and preterm delivery; and postpartum factors such as the baby’s birthweight and breastfeeding. We excluded studies with no relevant data, an inappropriate outcome, no original data (e.g. meeting abstract, editorial, commentary or letter) or duplicate data. We accepted the authors’ definitions of GDM and type 2 diabetes. Any discrepancies in selection were resolved by discussion with a third reviewer (ST).

### Data extraction and quality assessment

Two reviewers (GR and AAH) independently undertook study quality assessment and data extraction using predesigned forms. We used the Newcastle–Ottawa scale [23], which evaluates the representativeness and selection of studies, comparability among cohorts, ascertains the exposure and outcome, and evaluates the length and adequacy of follow-up. The risk of bias was considered to be low if a study obtained four stars for selection, two for comparability and three for follow-up; and medium if a study scored two or three stars for selection, one for comparability and two for follow-up. Studies scoring one or zero stars for selection and follow-up, and with no star for comparability, were deemed to have a high risk of bias [24]. Data were extracted as 2 × 2 tables for dichotomous outcomes, and as means and SEs for continuous outcomes.

### Data synthesis and analysis

For various risk factors, we calculated the RRs for dichotomous variables, and plotted point estimates and 95% CIs for progression to type 2 diabetes for women with GDM associated with various risk factors. For continuous variables, we plotted pooled mean differences with 95% CIs to assess differences between women with and without type 2 diabetes. We assessed the heterogeneity of association graphically with forest plots and statistically with χ^2^ tests and the *I*^*2*^ statistic. We pooled results using random effects models. To facilitate comparison between the strength of association of various risk factors with type 2 diabetes, we transformed the pooled standardised mean differences of continuous outcomes into RRs, assuming a 20% baseline risk of type 2 diabetes [25].

We specified the following subgroup analyses a priori: length of follow-up (≤1 year vs >1 year), ethnicity (white vs non-white), use of strict criteria to exclude possible pre-existing type 2 diabetes (such as GDM before 20 weeks of pregnancy or type 2 diabetes diagnosis in the first year after delivery; yes vs no). Additionally, we investigated differences between subgroups based on the levels of fasting glucose criteria for diagnosing GDM (<5.8 mmol/l vs ≥5.8 mmol/l). All analyses were performed using the Review Manager (RevMan Computer program, Version 5.2; The Nordic Cochrane Centre, Copenhagen, Denmark) [26].

## Results

### Study identification

**Fig. 1:**
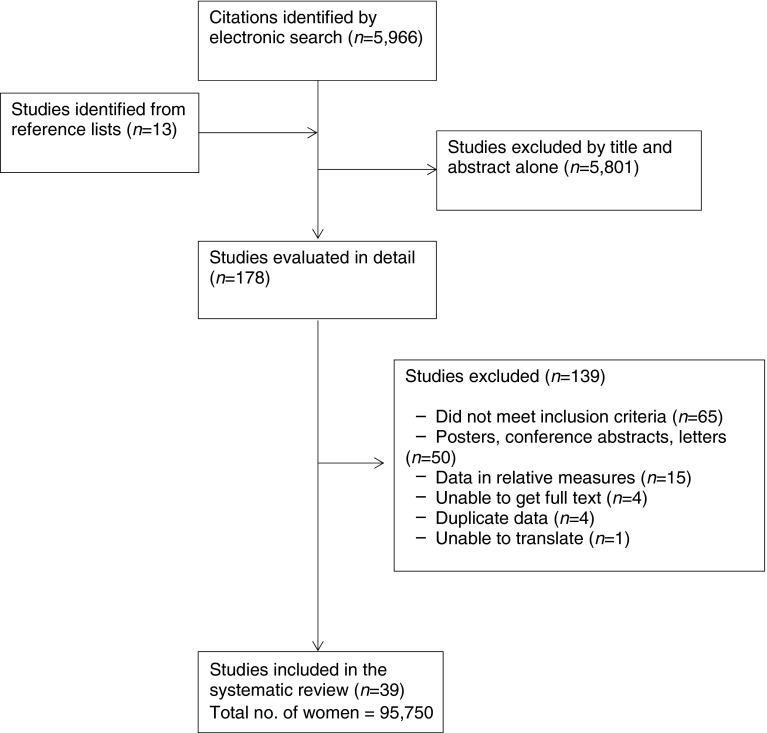
Flow chart for study selection for the systematic review of the predictors of progression to type 2 diabetes in women with GDM

From 5,966 citations, we selected 178 studies for further evaluation (Fig. 1). After a detailed assessment, we included 39 studies (95,750 women) [13–15, 17, 19–21, 27–58]. We excluded 139 studies for the following reasons: inclusion criteria were not met (*n* = 65); presented as posters, conference abstracts or letters (*n* = 50); inappropriate data format (*n* = 15); only abstracts were available (*n* = 4); duplicate data (*n* = 4); and could not be translated from the language of publication (*n* = 1).

### Characteristics and quality of the included studies

In all, there were 22 prospective cohort studies (56%) [13, 14, 17, 19–21, 29, 32, 35–37, 39–41, 43, 44, 46–48, 50, 52, 55] and 17 retrospective cohort studies (44%) [15, 27, 28, 30, 31, 33, 34, 38, 42, 45, 49, 51, 53, 54, 56–58]. The studies evaluated the association of maternal characteristics, pregnancy-specific factors and postpartum characteristics with progression to type 2 diabetes in women with GDM. The studies varied in their diagnostic criteria for both GDM and type 2 diabetes. Maternal characteristics including BMI, ethnicity and family history of diabetes were evaluated, as were risk factors specific to pregnancy such as maternal age at diagnosis of GDM, parity, gestational age at diagnosis, weight gain in pregnancy, hypertensive diseases in pregnancy and preterm birth, and levels of fasting, post-load blood glucose levels during OGTT, HbA_1c_ in pregnancy and use of insulin for managing GDM.

**Fig. 2:**
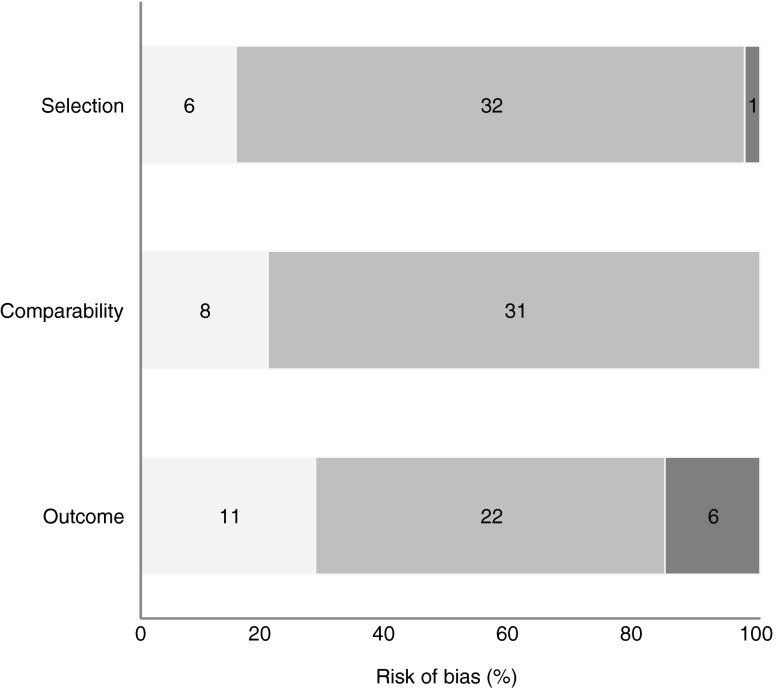
Risk of bias assessment on the Newcastle–Ottawa Scale for studies included in the systematic review of type 2 diabetes prediction in women with GDM. Light grey bars, low risk; mid grey bars, medium risk; dark grey bars, high risk. The numbers of studies are shown

The duration of follow-up varied from 6 weeks to 20 years after birth. A total of 29 studies (74%) evaluated the long-term risk of type 2 diabetes (>1 year after delivery) and 10 (26%) evaluated the risk of developing type 2 diabetes in the first year after childbirth. Detailed characteristics of the included studies are provided in ESM Table 1. In all, six studies (15%) had a low risk of selection bias, 32 (82%) had a medium risk and one (3%) had a high risk. Eight studies (21%) had a low risk of bias for comparability of cohorts, and 31 (79%) had a medium risk of bias. For outcome assessment, 11 studies (28%) had a low risk of bias, 22 (56%) had a medium risk and 6 (15%) had a high risk (see Fig. 2 and ESM Table 2).

### Maternal characteristics and progression to type 2 diabetes

**Fig. 3:**
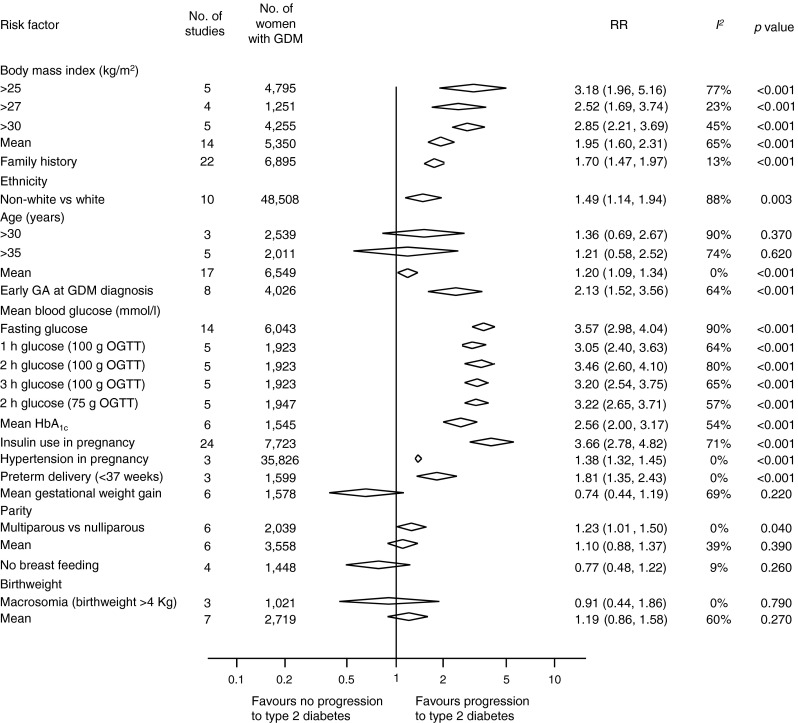
Summary estimates for the association of maternal risk factors with progression to type 2 diabetes in women with GDM. ‘Exclusively’ and ‘mostly’ breastfed were combined into a single breastfeeding category. ‘Mixed or inconsistent’ breastfeeding and ‘exclusively or mostly formula fed’ were combined into a single ‘no breastfeeding’ category [17]. Similarly, data reported for age <34 years in one study [37] was categorised as age <35 years, and data reported as BMI of <28 kg/m^2^ for one study [50] was classified as a BMI of <27 kg/m^2^ because all other studies used this cut-off value. GA, gestational age

A high BMI doubled the risk of future type 2 diabetes (RR 1.95 [95% CI 1.60, 2.31]; *I*^*2*^ = 65%), and the risk was increased in obese and overweight women for BMI thresholds of 25 kg/m^2^ (RR 3.18 [95% CI 1.96, 5.16]; *I*^*2*^ = 77%), 27 kg/m^2^ (RR 2.52 [95% CI 1.69, 3.74]; *I*^*2*^ = 23%) and 30 kg/m^2^ (RR 2.85 [95% CI 2.21, 3.69]; *I*^*2*^ = 45%). A family history of diabetes (RR 1.70 [95% CI 1.47, 1.97]; *I*^*2*^ = 13%), non-white ethnicity (RR 1.49 [95% CI 1.14, 1.94]; *I*^*2*^ = 88%) and older age (RR 1.20 [95% CI 1.09, 1.34]; *I*^*2*^ = 0%) were found to be significant risk factors for progression to diabetes after GDM. No increased risk was observed for individual age cut-offs of 30 and 35 years (Fig. 3).

### Pregnancy-specific factors and risk of future diabetes

Increased levels of fasting (RR 3.57; 95% CI 2.98, 4.04; *I*^*2*^ = 90%), 1 h (RR 3.05; 95% CI 2.40, 3.63; *I*^*2*^ = 64%), 2 h (RR 3.46; 95% CI 2.60, 4.10; *I*^*2*^ = 80%) and 3 h (RR 3.2; 95% CI 2.54, 3.75; *I*^*2*^ = 65%) blood glucose levels after OGTT and high HbA_1c_ were associated with an increased risk of future diabetes (RR 2.56; 95% CI 2.00, 3.17; *I*^*2*^ = 54%). Women who required insulin to manage GDM were more likely to develop type 2 diabetes (RR 3.66 [95% CI 2.78, 4.82]; *I*^*2*^ = 71%) compared with those managed without insulin (Fig. 3).

Multiparity was a significant risk factor compared with nulliparity (RR 1.23 [95% CI 1.01, 1.50]; *I*^*2*^ = 0%). Women with pregnancy complications such as hypertensive disease (RR 1.38 [95% CI 1.32, 1.45]; *I*^*2*^ = 0%) and preterm delivery (<37 weeks) (RR 1.81 [95% CI 1.35, 2.43]; *I*^*2*^ = 0%) were more likely to develop type 2 diabetes in the future. There were no differences in gestational weight gain (mean difference −0.83 kg [95% CI −2.18, 0.51]; *I*^*2*^ = 65%) between the two groups (ESM Fig. 1).

### Delivery and postpartum factors

The risk of developing type 2 diabetes was not associated with birthweight (RR 1.19 [95% CI 0.86, 1.58]; *I*^*2*^ = 60%), fetal macrosomia (RR 0.91 [95% CI 0.44, 1.86]) or breastfeeding (RR 0.77 [95% CI 0.48, 1.22]; *I*^*2*^ = 9%).

### Subgroup analysis

There were significant between-group differences based on follow-up time (<1 year vs >1 year) for risk factors such as fasting glucose (*p* = 0.04), BMI (*p* = 0.03) and insulin use (*p* = 0.006) and type 2 diabetes. We did not observe any between-group differences based on ethnicity and GDM diagnostic criteria (fasting glucose level of <5.8 mmol/l or ≥5.8 mmol/l) for predictors such as maternal age, BMI, family history of diabetes, need for insulin in pregnancy and the risk of type 2 diabetes. There was a significant difference between subgroups based on strict criteria for excluding possible pre-existing type 2 diabetes for fasting glucose as a predictor of type 2 diabetes (*p* = 0.02), but no differences were observed for associations with other predictors (Table 1).

**Table 1:** Subgroup analysis for progression to type 2 diabetes after GDM by follow-up time, ethnicity, timing of diagnosis and fasting glucose criteria for GDM diagnosis

Risk factor	Follow-up			Ethnicity				Strict criteria used to exclude pre-existing type 2 diabetes?^a^			Fasting glucose for GDM diagnosis		
	<1 year	≥1 year	*p* value	White	Non-white	Mixed	*p* value	Yes	No	*p* value	<5.8 mmol/l	≥5.8 mmol/l	*p* value
Age	1.27 [1.11, 1.45]	1.14 [0.97, 1.32]	0.29	1.29 [0.99, 1.64]	1.19 [0.97, 1.41]	1.20 [1.03, 1.39]	0.85	1.11 [0.81, 1.45]	1.15 [0.96, 1.38]	0.79	1.47 [0.92, 2.17]	1.15 [1.01, 1.29]	0.32
Fasting glucose	4.13 [3.32, 4.59]	3.13 [2.54, 3.65]	0.04	4.13 [1.43, 4.91]	3.34 [2.78, 3.83]	3.38 [2.54, 4.03]	0.80	2.40 [1.89, 2.94]	3.48 [2.76, 4.04]	0.02	–	–	–
BMI	1.56 [1.15, 2.04]	2.20 [1.89, 2.51]	0.03	2.11 [1.29, 3.05]	2.06 [1.66, 2.51]	1.74 [1.24, 2.33]	0.63	1.95 [1.47, 2.47]	2.38 [2.04, 2.74]	0.18	1.98 [1.39, 2.60]	1.87 [1.41, 2.40]	0.81
Family history	1.63 [1.19, 2.24]	1.74 [1.46, 2.07]	0.72	1.49 [1.20, 1.85]	1.78 [1.35, 2.35]	1.95 [1.45, 2.62]	0.31	1.80 [1.27, 2.54]	1.73 [1.39, 2.15]	0.86	1.83 [1.25, 2.66]	1.72 [1.42, 2.09]	0.78
Insulin	7.19 [4.14, 12.48]	3.06 [2.39, 3.92]	0.006	3.49 [2.25, 5.40]	4.09 [2.27, 7.34]	3.37 [2.43, 4.67]	0.85	2.62 [1.70, 4.02]	3.43 [2.62, 4.49]	0.29	4.37 [2.46, 7.76]	4.46 [3.63, 5.49]	0.95

Data are RR (95% CIs)

^a^Diagnosis of GDM before 20 weeks of gestation or type 2 diabetes less than 1 year after delivery

## Discussion

### Summary of findings

The future risk of diabetes appears to be mainly influenced by the gestational glycaemic status, and not by the mother’s gestational weight gain or baby’s birthweight. We found that both hypertensive disorders in pregnancy and preterm delivery in GDM pregnancies were associated with future onset of type 2 diabetes, which was previously unknown. Factors specific to pregnancy such as gestational age at onset of GDM and general maternal characteristics such as BMI, ethnicity and family history were also associated with future onset of type 2 diabetes. This systematic review has thus collated the information necessary for postnatal counselling of women with GDM.

### Strengths and limitations

To our knowledge, this is the first review to quantify the links between clinical characteristics (especially those relevant to pregnancy) and the future onset of diabetes in women with GDM. This systematic review used a prospective protocol and a comprehensive search without any language restrictions. Our meta-analysis included a large number of studies, and we were able to study most of the potentially relevant risk factors. Previous reviews were limited in their findings by the small number of studies included and the absence of summary estimates [2, 16, 18, 59]. We have provided clinically relevant estimates as RRs for use in counselling of women.

The studies included had varying definitions of population, risk factors and methods of ascertaining the outcome, which included different lengths of follow-up. Subgroup analysis was planned a priori to identify any sources of heterogeneity. It is possible that some studies could have misclassified pre-existing type 2 diabetes as GDM, especially when the diagnosis was made as early as 6 weeks after delivery. However, we did not observe significant between-group differences based on strict criteria for excluding pre-existing type 2 diabetes (studies that excluded women with GDM diagnosed within 20 weeks’ gestation or diabetes in the first year after delivery vs studies that did not). Some studies included only diet-controlled GDM, and this may have underestimated the risk of progression to type 2 diabetes.

The studies varied in the criteria used for diagnosing GDM and in the thresholds for commencing insulin in pregnancy; a third used Carpenter and Coustan criteria for diagnosing GDM. Our subgroup analysis was based on a fasting glucose cut-off value of 5.8 mmol/l: 69% of studies under this cut-off used Carpenter and Coustan criteria, and showed no significant differences in estimates for progression based on the criteria used for GDM diagnosis. Despite these variations, we consistently observed an increased risk of future diabetes in women with gestational hyperglycaemia.

We evaluated associations of individual predictors with outcomes, but could not adjust for confounding variables such as BMI because of the lack of access to individual data. The lack of detail in reported data made it difficult to evaluate the simultaneous influence of multiple factors on outcome. However, we could provide robust, precise estimates for individual risk factors that are relevant for providing postnatal information to women with GDM.

### Comparison with existing evidence

In the non-pregnant state, BMI, family history of diabetes and ethnicity are associated with the risk of type 2 diabetes [18, 60–63]. We observed the same in mothers with GDM, and there was a greater risk of progression in non-white than in white women. Individual studies have shown that particular ethnic groups such as blacks and South Asians are at an increased risk [15, 34]. Owing to the various classifications of ethnicity used in primary studies, we were unable to provide progression rates for specific ethnic subgroups, which may have contributed to the heterogeneity.

We did not identify an increased risk for women with high weight gain during pregnancy. However, it is likely that women with severe GDM undergo intensive monitoring and receive significant input regarding their diet and lifestyles, thereby restricting their weight gain in pregnancy [50].

Although previous studies have shown an improvement in glucose homeostasis with breastfeeding [17, 64], we found no significant association between the absence of breast-feeding and progression to diabetes. This could be attributed to the imprecise estimates obtained owing to the small number of studies and individuals. Previous studies have shown improved glucose values on OGTTs, especially in the short term [64]. Our review measured the impact on type 2 diabetes and not on lesser degrees of glucose intolerance. Furthermore, the beneficial effect on glucose levels may not have been sustained in the long term. The rigour with which breast-feeding information was collected also varied by study; most data were self-reported by the women.

In current practice, the criteria used for diagnosing GDM are not applicable in many centres. However, given the long-term outcomes evaluated in our review, we were unable to assess for heterogeneity in estimates based on the WHO 2013 criteria for diagnosing GDM [65], which are recommended in many existing national and international guidelines.

### Implications for clinical practice

Pregnancy is an important point in the life of a woman when she has regular contact with the healthcare system, thus providing opportunities to influence the future health of both mother and child. One of the major factors responsible for poor postnatal screening for diabetes and subsequent follow-up has been the lack of clear communication between secondary and primary care providers [10, 66]. Pregnancy-specific findings such as glycaemic control, despite being associated with a fourfold increase in the risk of future diabetes, are not taken into count during counselling.

Postnatal advice to women with GDM should incorporate information on their individual risk factors. Communication between hospitals and general practitioners on the mother’s risk of future diabetes could be improved by providing discharge summaries with pregnancy-specific details such as OGTT results, gestational age at GDM diagnosis, use of insulin, and complications such as pre-eclampsia and preterm birth. An efficient system of data linkage and communication between secondary and primary care providers will enable general practitioners to incorporate this information into their management of mothers; in particular, the management of women at a high-risk of developing type 2 diabetes could involve measures such as reminder systems [67]. Women are more likely to comply with diet and lifestyle changes if they know their individual risks of future diabetes [68].

### Research recommendations

Current prediction models for type 2 diabetes should be updated and validated by including the factors identified in this review. There is a need for well-designed, prospective long-term studies to explore the association between breastfeeding and future type 2 diabetes [64]. The meta-analysis of individual participant data (IPD) could overcome many of the limitations of our meta-analysis [69]. A large-scale IPD meta-analysis could enable us to predefine the desired clinically relevant endpoints (e.g. timing of diabetes onset) and cut-off values for clinical variables, standardise the definitions of predictors and outcomes, take into account the performance of many candidate prognostic variables, directly handle missing data on both predictors and outcomes, and account for heterogeneity in baseline risks.

### Conclusion

Postnatal counselling of women with GDM should be individualised for the risk of future diabetes.

## Electronic supplementary material

Below is the link to the electronic supplementary material.

ESM Fig. 1 (PDF 149 kb)

ESM Table 1 (PDF 182 kb)

ESM Table 2 (PDF 181 kb)

ESM PRISMA checklist (PDF 206 kb)

ESM Search strategy (PDF 51 kb)

## Abbreviations

GDM: Gestational diabetes mellitus
IPD: Individual participant data
PRISMA: Preferred Reporting Items for Systematic Reviews and Meta-Analyses
