# Supplementary material for: Quantification of the type 2 diabetes risk in women with gestational diabetes: a systematic review and meta-analysis of 95,750 women
Source: Diabetologia. 2016 Apr 13;59:1403–11. doi: 10.1007/s00125-016-3927-2 (PMC4901120; doi:10.1007/s00125-016-3927-2)
Supplement: Supplementary file 1 — (PDF 149 kb) [file 125_2016_3927_MOESM1_ESM.pdf]

ESM Figure 1 Summary estimates of association of maternal risk factors (continuous variables) and progression to type 2 diabetes in women with gestational diabetes

Risk factor	No. of studies	No. of women with gestational diabetes	Mean difference	I^2	*p* value
Body mass index (kg/m^2)	14	5,350	2.14 [1.56, 2.72]	42%	< 0.00001
Age (years)	17	6,549	0.59 [0.23, 0.94]	0%	0.001
GA at diagnosis (weeks)	8	4,026	-2.88 [-4.25,-1.50]	70%	< 0.0001
Blood glucose (Mmol/l)					
Fasting glucose	14	6,043	1.21 [0.85, 1.57]	88%	< 0.00001
1-h glucose (100 g OGTT)	5	1,923	1.84 [1.09, 2.60]	69%	< 0.00001
2-h glucose (100 g OGTT)	5	1,923	2.51 [1.65, 3.38]	64%	< 0.00001
3-h glucose (100 g OGTT)	5	1,923	2.35 [1.89, 2.81]	5%	< 0.00001
2-h glucose (75 g OGTT)	5	1,947	1.69 [1.16, 2.22]	48%	< 0.00001
HbA_{1c} (%)	6	1,545	0.46 [0.22, 0.70]	80%	0.0002
Gestational weight gain (Kg)	6	1,578	-0.83 [-2.18, 0.51]	65%	0.23
Parity	6	3,558	0.06 [-0.15, 0.28]	48%	0.58
Birth weight (Kg)	7	2,719	0.07 [-0.06, 0.19]	61%	0.29

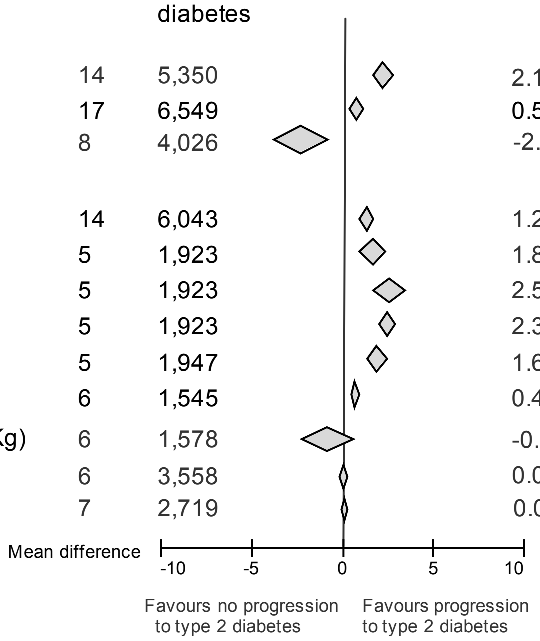

GA gestational age
