# Supplementary material for: Quantification of the type 2 diabetes risk in women with gestational diabetes: a systematic review and meta-analysis of 95,750 women
Source: Diabetologia. 2016 Apr 13;59:1403–11. doi: 10.1007/s00125-016-3927-2 (PMC4901120; doi:10.1007/s00125-016-3927-2)
Supplement: Supplementary file 2 — (PDF 182 kb) [file 125_2016_3927_MOESM2_ESM.pdf]

ESM Table 1 Characteristics of the studies included in the systematic review on quantification of the type 2 diabetes risk in women with gestational diabetes mellitus

Study year	Country	Study design	*n* (women with GDM)	Inclusion criteria and testing for GDM	Exclusion criteria	Risk factors studied	Outcome
Kwak 2013	South Korea	Prospective cohort	843	Women with GDM enrolled from a hospital in a defined period. Universal screening for GDM by a 1-h 50-g GCT followed by a 3-h 100-g OGTT if glucose value ≥ 7.2 mmol/l. GDM was diagnosed by the Third International Workshop-Conference on GDM	Women who had diabetes before pregnancy or if they were positive for GADA	Age at pregnancy, pre-pregnancy BMI, parity, gestational weight gain, gestational age at diagnosis, family history of diabetes, treatment with insulin, glucose values for 100-g OGTT during pregnancy, genetic variants in 10 known T2D associated genes	Women were tested for T2D with 75-g OGTT (ADA 2012 criteria) at 2 months postpartum and annually for a median of 49 months (IQR 30-82 months)
Wang 2012	United States	Retrospective cohort	1,142	All women aged 13-50 years with first record of pregnancy in the LSUHCSD (Louisiana State University Health Care Services Division) database between 1990 and 2009. GDM was diagnosed by WHO (1999) or ADA (1997, 2003) cut-offs for fasting glucose, 75-g or 100-g OGTT, or random glucose	Women with pre-existing diabetes and women with missing information	Age, BMI, race (African American, white, Asian, Hispanic and Indian), follow-up time after delivery	Follow-up information was obtained though a database. Follow-up for a mean of 8.6 years (1990-2009). ADA (2003), WHO (1998) criteria were used for diagnosis of T2D
Tura 2012	Italy	Prospective cohort	76	Women with GDM recruited from outpatient department of a hospital. GDM was diagnosed according to the criteria of the Fourth Workshop Conference of Gestational Diabetes	a) Women who had developed diabetes at first analysis (early postpartum)<br>b) Follow-up less than three years unless they developed diabetes within that period<br>c) Further pregnancies	Baseline values at 4-6 months postpartum including age, BMI, glucose values in 75-g OGTT, insulin and C-peptide levels, blood pressure, lipids, family history of diabetes and insulin use during pregnancy	Women were tested for T2D at 4-6 months post-partum and annually for 7 years. ADA (2003) criteria for T2D

					during the follow-up period d) If glucose, insulin and C-peptide during an OGTT at each evaluation were not available		
Gunderson 2012	United States	Prospective cohort	522	KNPC (Kaiser Permanente Northern California) members with GDM who were enrolled into Study of Women, Infant Feeding, and Type 2 Diabetes (SWIFT) cohort and had a singleton, live birth ≥35 weeks' gestation and were able to provide information on duration and intensity of breast-feeding and formula feeding. GDM was diagnosed by ADA (2000) criteria for 3-h 100-g OGTT at 24-32 weeks gestation	Pre-existing major medical conditions prior to index GDM pregnancy	Lactation intensity (comparison of the effect of breast-feeding and formula feeding on postpartum glucose, insulin and insulin resistance/sensitivity)	Women were tested for T2D at 6-9 weeks post partum by 2-h 75-g OGTT (ADA criteria)
Chew 2012	Malaysia	Prospective cohort	342	Women aged 20–50 years were recruited by a systematic random sampling from the hospital's database of women with GDM. GDM was diagnosed by WHO (1985) criteria	a) Women currently pregnant<br>b) Those who had progressed to T2D at the time of recruitment	FH of T2D in first degree relatives, macrosomia, infant's birth weight, previous pregnancies with GDM, duration lapse after GDM pregnancy, lipid profile and OGTT during index GDM pregnancy and at the time of study, body weight and blood pressure at diagnosis of index GDM and at the time of study, insulin use for GDM during pregnancy, age and ethnicity	75g OGTT (WHO 2002) between 3 months -15 years postpartum
Xiang 2011	United States	Retrospective cohort	12,998	Women who had a singleton delivery at ≥ 20 weeks gestation in KPSC (Kaiser Permanente Northern	Pre-existing diabetes	Ethnicity (Non Hispanic White, Hispanic, Black, API, others) as risk factor for T2D in women with/without GDM. Also GDM	Followed up to 15 years. Diagnosed as T2D by<br>a) ADA (2010)

				California) hospitals during a defined period with at least 1 year of continuous health plan membership before the index pregnancy and after delivery. Gaps in membership up to 90 days were allowed. GDM was diagnosed by at least two values ≥ Carpenter Coustan thresholds on a 3-h 100-g or 2-h 75-g OGTT; or a single value of 1-h 50-g GCT ≥ 11.1 mmol/l		as a risk factor for future diabetes	criteria<br>b) HbA_{1c}≥7% (53 mmol/mol)<br>c) Prescription of insulin or other glucose lowering drugs<br>d) ICD coding of hospital encounters
Kim 2011	South Korea	Prospective cohort	381	Women with GDM attending a hospital in a defined period. GDM was diagnosed by universal screening with 50-g GCT followed by Carpenter Coustan thresholds for 3-h 100- OGTT	Pre-existing diabetes, GDM with GADA	Age, height, income, job, BMI pre-pregnancy and at delivery, parity; FH of diabetes; fasting glucose, insulin, c-peptide, insulin resistance, lipids, HbA_{1c}, blood pressure, CRP during pregnancy and postpartum; insulin use in pregnancy, insulin dose, GWG, GA at delivery, baby weight, breast-feeding, post-partum energy expenditure and nutrient intake	OGTT at 6-12 weeks postpartum. T2D diagnosed by ADA (2004) criteria
Gobl 2011	Austria	Prospective cohort	110	Women with GDM attending a diabetes outpatient clinic during pregnancy, recruited within 3-6 months after delivery. GDM was diagnosed as per criteria of Fourth International Workshop Conference on Gestational Diabetes	Women with overt T2D at the time of recruitment (3-6 months post partum)	Age, BMI, waist circumference, OGTT, insulin resistance, triglyceride, HDL and blood pressure at study baseline; ethnicity, FH of T2D and insulin use in pregnancy	Women were tested for T2D at recruitment (3-6 months) and then annually up to 10 years. Diabetes diagnosed if fasting or 2-h glucose exceeded 126 mg/dl or 200 mg/dl

Ekelund 2010	Sweden	Prospective cohort	144	GDM women referred to a hospital in a defined time period. GDM was diagnosed by a 2-h capillary blood glucose value ≥ 9 mmol/l on a 75-g OGTT	Women who were unable to undergo repeat OGTT at study start	Age, BMI, FH of diagnosis, HbA_{1c}, previous pregnancy, GA at diagnosis, birth weight, ethnicity, insulin treatment, insulin resistance, preterm delivery, GADA, OGTT glucose and insulin levels during pregnancy; BMI, waist-hip ratio, HbA_{1c}, lipids, OGTT glucose and insulin during interim follow-up	Tested for T2D with WHO (1999) criteria at one, two and five years post partum
Feig 2013	Canada	Retrospective cohort	34,428	All women aged 15 to 50 years from a discharge database who delivered in an Ontario hospital. For women with multiple deliveries, one pregnancy was chosen as index randomly. GDM was diagnosed by diagnostic code for diabetes in hospitalisation record during delivery or by ≥ 3 outpatient diabetes-related medical claims up to 4 months leading to delivery	a) Pre-existing diabetes<br>b) Non-residents of Ontario<br>c) Those having less than two years of health plan coverage prior to baseline	Preeclampsia, gestational hypertension, gestational diabetes and combination of gestational diabetes with preeclampsia/gestational hypertension	Followed up from 180 days postpartum to 16.5 years (mean 8.5 years) for the development of diabetes, evidenced by a new record in the Ontario Diabetes Database
Ogonowski 2009	Poland	Prospective cohort	318	White women older than 18 years diagnosed with glucose intolerance during pregnancy referred to an outpatient clinic in a defined period. GDM was diagnosed through 2-step procedure at 24-28 weeks gestation by a GCT followed by OGTT and WHO (1999) criteria were used	Women with previous diabetes or multiple pregnancies	Age, BMI, glucose values in GCT and OGTT, HbA_{1c}, insulin treatment, insulin dose during pregnancy and glucose values in OGTT at follow-up	WHO (1999) criteria for T2D at 6 weeks post partum
Hossein-Nezhad 2009	Iran	Prospective cohort	98	All pregnant women consecutively referred to 5 university hospitals were	-	Pre-pregnancy BMI, age, GA at diagnosis of GDM, parity, insulin treatment during	ADA criteria for diagnosis of diabetes at 6-12 weeks

				recruited. GDM was diagnosed through universal screening at 24-28 weeks gestation by a 1-h 50-g GCT followed by 3-h 100-g OGTT with Carpenter Coustan criteria		pregnancy, fasting plasma glucose during pregnancy, FH of diabetes, history of abortion/macrosomia	postpartum
Russell 2008	Canada	Retrospective cohort	1,401	All GDM women who presented their first record of pregnancy in the Nova Scotia Atlee Perinatal Database (NSAPD) in a defined time period. GDM was diagnosed by universal screening at 24-28 weeks gestation by a GCT ± OGTT with diagnostic criteria as per Canadian Diabetes Association guidelines	Women with pre-existing diabetes; subjects diagnosed within the first year after the index pregnancy were excluded from the risk factor analysis, but were included for reporting rates of diabetes by follow-up time	Age, pre-pregnancy and delivery weight, insulin treatment for GDM, chronic hypertension, endocrine disease, PIH, BW, gestational age at delivery, apgar score, neonatal hypoglycaemia, recurrent GDM	Development of diabetes was verified by hospital discharge summary with diagnostic code for diabetes, or outpatient physician service claims. Follow-up was up to 13 years postpartum
Rivero 2008	Brazil	Prospective cohort	109	All women with potential GDM who had given birth (over 1 year earlier) referred to a hospital in a defined time period. GDM diagnosis was confirmed with a 100-g OGTT by ADA (1997) or 75-g OGTT by ADA (2004) criteria	a) Gastrointestinal problems after glucose loading<br>b) Women who had incomplete screening<br>c) Women with diabetes 6 weeks after delivery<br>d) Those who were subsequently found not to have GDM	Age, parity, BMI pre-pregnancy, BMI at follow-up, blood pressure, lipids, fibrinogen, hsCRP and microalbuminuria at follow-up	Mean follow-up 32 ± 15 months and T2D diagnosed by ADA (2004) criteria
Krishnaveni 2007	India	Prospective cohort	35	All pregnant women during a defined period at a hospital. GDM was diagnosed with a 100-g OGTT using Carpenter Coustan criteria	Pre-existing diabetes	Insulin treatment, parity 2+, fasting glucose, insulin levels and insulin resistance during pregnancy; age, BMI, height, waist-hip ratio, skin fold thickness, insulin resistance and FH of diabetes at follow-up	WHO (1999) criteria for T2D at 5 years

Oldfield 2007	England	Prospective cohort	73	Women diagnosed with GDM during a defined time period. Women with risk factors were screened and WHO (1980) criteria for 75-g OGTT used for GDM diagnosis	Women with GDM diagnosed before 20 weeks	Age during pregnancy and at follow-up. glucose values during antenatal OGTT, BMI, FH of diabetes, HbA1c and insulin use during pregnancy	Diabetes was diagnosed using WHO (1998) criteria
Schaefer-Graf 2002	United States	Retrospective cohort	1,636	Women followed up for GDM in a hospital during a defined time period. GDM was diagnosed by universal screening with a 2-step procedure with the criteria defined by the Third International Conference Workshop on GDM	Incomplete antepartum data	Age, fasting glucose, glucose value for GCT, area under the glucose curve for OGTT during pregnancy; BMI before pregnancy and after delivery; previous macrosomia, still birth, congenital anomaly, parity and GDM; birth weight, gestational age at OGTT	Fasting glucose was measured before discharge, and women who needed insulin were classified as having diabetes. 75-g 2-hour OGTT was scheduled for others between 1 and 4 months after delivery and diabetes diagnosed using the 1979 National Diabetes Mellitus Data Group criteria
Zonenberg 2006	Poland	Prospective cohort	84	Women treated for GDM during a defined time period in a diabetes clinic. GDM diagnostic criteria not reported	-	Age; BMI during pregnancy and at follow-up; insulin use during pregnancy; parity, birth weight; OGTT glucose and insulin, insulin resistance, C-peptide, HbA_{1c}, lipids and leptin at follow-up	Polish Diabetes Association guidelines used for diagnosing T2D. Followed up to 5 years after GDM
Kousta 2006	England	Prospective cohort	368	Women with previous GDM and belonging to any of the following ethnicities: European, Asian-Indian (India, Pakistan, Bangladesh or Sri Lanka) or African-Caribbean recruited from a database. GDM diagnosed by WHO (1999) criteria		Ethnicity; age, BMI, waist circumference, blood pressure, lipids, fasting glucose, insulin level, beta cell function and insulin sensitivity at follow-up	75-g OGTT (WHO criteria) was used to test for diabetes at 20 months (18.2-22.1 months) post-partum for 62% participants. Remainder underwent fasting glucose

Jarvela 2006	Finland	Retrospective cohort	435	Women with singleton pregnancy diagnosed with GDM for the first time who delivered at a hospital during a defined time period. GDM was diagnosed by selective screening of high-risk patients through a 2-h 75-g OGTT with glucose values as per criteria defined by Finnish Diabetes Association	-	Age, insulin use in pregnancy, autoantibodies in pregnancy including GADA, ICA, IAA, IA-2A	Based on questionnaire information and use of medications for diabetes. Mean follow-up period from delivery to the date of completing the questionnaire was 5.7 years
Cho 2006	South Korea	Prospective cohort	909	Women with GDM followed-up in four major hospitals in Korea during a defined period. GDM was diagnosed by a 50-g screening test at 24-28 weeks gestation followed by a 3-h OGTT at 28-32 weeks with NDDG criteria	None stated	Family history, parity, pre-pregnancy body weight, waist/hip ratio, postpartum BMI, body fat %, skin-fold thickness, lipid profile, insulin resistance, occupation	2-h 75-g OGTT with NDDG criteria was used for diagnosis of diabetes. Mean postpartum follow-up period was 2.13 +/-1.75 years (up to 6 years postpartum)
Cheung 2006	Australia	Prospective cohort	102	Women followed-up during GDM at two different hospitals in Sydney during a defined period. GDM was diagnosed by universal screening with 50-g GCT followed by 2-h 75-g OGTT at one hospital, and selective screening of at-risk women with 2-h 75-g OGTT directly in another hospital. Local criteria for diagnosis were used	When diagnosis of diabetes could not be confirmed and the patient refused to be retested	Age at conception, fasting glucose, 75g OGTT values in pregnancy, BMI in early pregnancy, FH, insulin use in pregnancy including timing and duration, and parity	WHO criteria were used. Follow-up was 0-8 years (mean 4.5 years)

Pallardo 1999	Spain	Retrospective cohort	788	White women with a singleton pregnancy followed up for GDM in a hospital during a defined time period. GDM was diagnosed by a 2-step process with a 1-h 50-g GCT followed by a 2-h 75-g OGTT with NDDG criteria. Universal screening was performed.	Women who did not attend the initial postpartum metabolic assessment	Age, fasting glucose, 100g OGTT glucose values in pregnancy, fasting and postprandial c-peptide in pregnancy, pre-pregnancy BMI, FH of diabetes, insulin use in pregnancy, parity, third trimester HbA_{1c}, macrosomia, gestational age at diagnosis of GDM, previous macrosomia/ congenital malformations/ perinatal mortality/preterm delivery, previous GDM	WHO (1985) criteria and the ADA (1997) criteria were used. Follow-up was 4.2 ± 1 months post-partum
Buchanan 1999	United States	Prospective cohort	91	GDM was diagnosed by a 2-step process, a 50-g GCT and a 3-h 100-g OGTT as per criteria of Third International Workshop Conference on Gestational Diabetes. All Latino women referred for management of GDM at a hospital were invited if they met all of the following criteria:<br>a) Gestational age of 28-34 weeks<br>b) No current/previous insulin therapy<br>c) All fasting serum glucose values <7.2 mmol/l<br>d) Uncomplicated singleton pregnancy and<br>e) Both parents and at least three of four grandparents from Guatemala, Mexico or El Salvador.	Women with persistent diabetes at 6 months, and women whose post-partum diabetes status could not be determined	During pregnancy - Age, fasting glucose, blood pressure, body fat %, subscapularis/triceps skin-fold thickness ratio, gestational weight gain, gestational age at diagnosis of GDM and at study entry. At final follow up – BMI, breast-feeding, weight change from initial postpartum weight	WHO criteria were used.<br>Follow-up was between 11 and 26 months (median 15.4 months) postpartum.

Dalfra 2001	Italy	Prospective cohort	70	Consecutive women with GDM identified through universal screening of pregnant women in an antenatal clinic during a defined time period. GDM was diagnosed by Carpenter and Coustan criteria through a 2-step screening process	Women who developed T1D	Mean age in pregnancy, fasting glucose and glucose values under OGTT in pregnancy and at one year postpartum, 3rd trimester HbA_{1c}, BMI before pregnancy and at one year postpartum, FH, insulin use in pregnancy, macrosomia, birth weight, gestational weight gain, gestational age at diagnosis of GDM	WHO (1990) criteria were used for diagnosis of diabetes and follow-up was for up to 5 years postpartum
Steinhart 1997	United States	Retrospective cohort	111	Navajo women with gestational diabetes who received prenatal care in a hospital during a defined period. GDM was diagnosed by screening with 1-h 50-g GCT followed by a 3-h 100-g OGTT as per American College of Obstetrics and Gynecology (1994) guidelines	Women who only had one elevated plasma glucose level on a OGTT, non-Navajo lineage, a diagnosis of diabetes before the index pregnancy and lost or poorly documented records	Mean age in pregnancy, parity, infant weight, fasting glucose and total OGTT value in pregnancy, insulin use in pregnancy, spontaneous abortion, recurrent GDM	WHO (1985) criteria were used for diagnosis of diabetes. Follow-up was for 6-11 years postpartum
Damm 1992	Denmark	Prospective cohort	241	All women followed up for GDM at a hospital during a defined time period. GDM was diagnosed by local criteria with a 3-h 50-g OGTT. Screening was based on risk factors and fasting glucose values	Women whose GDM was treated by oral hypoglycaemic agents or insulin	Ethnicity, age in pregnancy, pre-pregnancy BMI, FH of diabetes, parity, GA at diagnostic OGTT and at delivery, preterm delivery, LGA	WHO (1985) criteria were used. Follow-up was for 2-11 years postpartum
Weijers 2006	Netherlands	Retrospective cohort	168	All pregnant women referred to a teaching hospital during a defined period were screened with a 2-step protocol with 1-h 50-g GCT and 3-h 100-g OGTT at initial visit. GDM was diagnosed by criteria as per Fourth International Workshop -Conference on	Polycystic ovary syndrome, hyperthyroidism, or pre-pregnancy diabetes	Age, ethnicity, FH of diabetes, obstetric history, parity, macrosomia, PIH, apgar score, fasting c-peptide and glucose, glucose values in GCT and 3-h OGTT during pregnancy	ADA (1997) criteria were used. Followed up to 6 months postpartum

				Gestational Diabetes Mellitus			
Hunger-Dathe 2006	German	Prospective cohort	173	Women followed up for GDM at a hospital during a defined time period. Criteria for GDM diagnosis not reported	Women of non-white origin	Age at first consultation, education, fasting glucose, OGTT glucose values in pregnancy, BMI pre-pregnancy and at follow-up, FH, insulin use, gestational age at diagnosis, blood pressure and HbA_{1c} at reevaluation, weight gain after delivery	German Diabetes Society 2002 criteria were used. Follow-up was 5.8 +/-2 (2-10) years
Mukerji 2012	Canada	Retrospective cohort	33,203	All women aged 20–49 years, residing in Ontario and using state-funded health insurance program with in-hospital live births during a defined period. Diagnosis of GDM was based on coding in hospitalisation record	Women with pre-existing diabetes, or missing address information	Ethnicity and GDM status	Diagnosis of post-partum diabetes on the basis of entry into the Ontario Diabetes Database (ODD). Median follow-up time of 7.6 years
Greenberg 1995	United States	Retrospective cohort	94	Women with GDM cared-for in a hospital during a defined period. Screening for GDM was by 1-h 50-g GCT at 24-28 weeks (earlier if risk factors) followed by 3-h 100-g OGTT. The Second International-Workshop Conference on GDM and Carpenter and Coustan criteria were used for diagnosing GDM	-	Antepartum variables including age, fasting glucose, 100-g OGTT values, BMI, insulin dose and timing of initiation, HbA_{1c} and gestational age at diagnosis; caesarian section, shoulder dystocia and birth weight percentile	The National Diabetes Data group criteria were used for diagnosing diabetes. Follow-up was at 6 weeks postpartum
Lin 2005	Taiwan	Prospective cohort	127	Women diagnosed with GDM in a hospital during a defined time period. GDM was diagnosed at 24-28 weeks by a 1-h 50-g GCT followed by a	Women with diagnosis of diabetes before pregnancy	During pregnancy- fasting glucose, 100g OGTT values, pre-pregnancy BMI, FH, gestational weight gain. Postpartum - age, BMI, waist/hip ratio, blood	ADA (1997) criteria were used for diagnosis of diabetes. Follow-up was at 1.0 to 19.0 (mean 3.3)

				3-h 100-g OGTT using Carpenter and Coustan criteria		pressure, lipid profile, HbA_{1c}, C-peptide, insulin resistance	months after delivery
Capula 2014	Italy	Retrospective cohort	454	All consecutive white women with singleton pregnancy diagnosed with GDM and who underwent a 2-h 75-g OGTT at 6-12 weeks postpartum. Testing for GDM was by OGTT at 24-28 weeks and diagnosis was as per Carpenter and Coustan and IADPSG criteria during different times of the study period	Pre-existing diabetes; untreated endocrinopathies; use of medications affecting glucose tolerance during postpartum testing; pregnancy at the time of postpartum OGTT	Age at pregnancy, gravidity, pre-pregnancy and postpartum BMI, FH, fasting glucose in pregnancy, insulin use in pregnancy, breast-feeding, previous GDM, PCOS, GA at diagnosis of GDM	ADA (2009) criteria were used. Follow-up was at 6-12 weeks postpartum
Bentley-Lewis 2014	United States	Retrospective cohort	124	Participants were selected from pathology specimens received in Massachusetts General Hospital in a defined period. They were then matched with clinical records and women with GDM diagnosed by Carpenter-Coustan criteria were included. Only those with full-term, singleton live births were included	Those who did not identify their race/ethnicity	Placental histomorphometry variations between white and non-white women with GDM. Also ethnicity-based comparison of clinical features such as age, BMI, blood pressure, lipid profile, preeclampsia, hypertension, smoking, birth weight and progression to type 2 diabetes	Development of T2D as per clinical database (diagnostic criteria not defined). Mean follow-up was 4.1 years (0-8.9 years)

Liu 2014	China	Retrospective cohort	1,263	Women in six urban districts diagnosed with GDM by WHO (1999) criteria and who participated in the Tianjin GDM prevention program were studied at 1-5 years postpartum. Women underwent universal screening for GDM by 1-h 50-g GCT at 26-30 weeks gestation, followed by 2-h 75-g OGTT if indicated	Diabetes at the time of recruitment, fetal death	Age during pregnancy, pre-pregnancy BMI, fasting and 2-h glucose on 75-g OGTT during pregnancy, insulin use in pregnancy, family history of diabetes, postpartum BMI, weight change from pre-pregnancy to postpartum, education, income, smoking, alcohol intake, leisure time physical activity, dietary intake	ADA (2005) criteria were used. Follow-up was at 1-5 years post-partum
Lin 2015	Taiwan	Retrospective cohort	130	Women with gestational diabetes were identified using hospital tracking system and coding in International classification of disease 9th revision, clinical modification (ICD-9-CM diagnostic code 648). The GDM diagnosis was based on a 1-h plasma glucose level of 7.8 mmol/L on the 50 g GCT, followed by at least two abnormal values on a 100 g OGTT as per NDDG criteria	Duplicate charts, missing charts, delivery outside study medical centre, previous history of type 1 or type 2 diabetes and women with no documented history of GDM	Age during pregnancy, family history of diabetes, parity, pre-pregnancy BMI, glucose values during GCT and OGTT, insulin use in pregnancy, gestational weight gain, mode of delivery and infant birth weight	ADA 2010 criteria were used. Follow-up was up to 9 years. 55% (n=71) underwent tests for diabetes and follow-up was up to 9 years.
Bao 2015	United States	Prospective cohort	1,695	Women who reported incident gestational diabetes between 1991 to 2001 in Nurses' Health Study II. GDM was self-reported. A validation study showed that 94% of self-reported GDM cases were confirmed by medical records (diagnostic criteria not reported).	Prevalent GDM before start of study; history of type 2 diabetes, cardiovascular disease or cancer; multiple gestation	Body mass index (cut-offs of 25, 30, 35 and 40) at baseline and follow-up; weight change from baseline	Type 2 diabetes self-reported using ADA (1997) criteria. NDDG criteria were used prior to 1998

Eades 2015	Scotland, UK	Retrospective cohort	164	Routinely collected data were used. All women were screened for GDM by fasting or random glucose at 28 weeks. A 75- g OGTT was performed if results were above thresholds (fasting glucose > 5.5 mmol/l, random glucose > 7.0 mmol/l within 2 hours of food or > 5.5 mmol/l after 2 hours); or if glycosuria present; or if there was family history of diabetes; or pregnancy was high-risk. GDM was diagnosed if fasting glucose > 5.5 mmol/l or 2-h glucose > 9.0 mmol/l on OGTT	GDM diagnosed in first trimester, known type 1 or type 2 diabetes before pregnancy	Deprivation categories, previous GDM, family history of diabetes; age, weight, fasting glucose, 2-h glucose and HbA_{1c} at diagnosis of gestational diabetes; insulin use in pregnancy and average weekly gain in pregnancy	T2D was confirmed by information in Scottish Case Information – Diabetes Collaboration (SCI-DC), a validated diabetes clinical information system. WHO 1999 criteria were used for 97% of cases with the rest being diagnosed as per WHO 1985 criteria
Carvalho Ribeiro 2015	Portugal	Retrospective cohort	300	Women born before 1995 who were diagnosed with GDM between 2001 and 2010 and received antenatal care in a hospital in Braga were randomly selected using computer generated software. GDM was diagnosed by OGTT, criteria not reported	Women born after 1995, incomplete data, delivery not in the same hospital	Pre-pregnancy BMI cut-off 26.4, postpartum BMI, family history of type 2 diabetes, previous pregnancy, previous GDM, GA < 24 weeks at diagnosis of GDM, insulin requirement in pregnancy, maternal age at diagnosis of GDM, fasting glucose	Data from a register of diabetes (in 2011) of all primary care centers in the area, criteria for diagnosis of type 2 diabetes not reported

Abbreviations: GADA glutamic acid decarboxylase antibodies, ICA islet cell autoantibodies, IAA insulin autoantibodies, IA-2A insulinoma-2-associated autoantibodies, IQR interquartile range, FH family history, GA gestational age, API Asian Pacific Islanders, GWG gestational weight gain, LGA Large for gestational age, PIH pregnancy induced hypertension, BW birth weight, hsCRP high-sensitivity c-reactive protein, NDDG National Diabetes Data Group, T1D type 1 diabetes, T2D type 2 diabetes, IADPSG International Association of the Diabetes and Pregnancy Study Groups
