# Supplementary material for: Quantification of the type 2 diabetes risk in women with gestational diabetes: a systematic review and meta-analysis of 95,750 women
Source: Diabetologia. 2016 Apr 13;59:1403–11. doi: 10.1007/s00125-016-3927-2 (PMC4901120; doi:10.1007/s00125-016-3927-2)
Supplement: Supplementary file 3 — (PDF 181 kb) [file 125_2016_3927_MOESM3_ESM.pdf]

ESM Table 2 Newcastle-Ottawa Scale rating of quality of included studies in the systematic review on quantification of the type 2 diabetes risk in women with gestational diabetes

	Selection						Comparability			Outcome				
**Study id/year**	Representativeness of the exposed cohort	Selection of the non-exposed cohort	Ascertainment of exposure	Outcome not present at the start	Total	Risk of bias	Comparability of cohorts on basis of design or analysis	Total	Risk of bias	Assessment of outcome	Follow-up long enough for outcome	Adequacy of follow-up	Total	Risk of bias
Kwak 2013	*	*	*	*	4	Low	*	1	Medium	*	*		2	Medium
Wang 2012		*	*	*	3	Medium	**	2	Low	*	*		2	Medium
Tura 2012		*	*		2	Medium	*	1	Medium	*	*	*	3	Low
Gunderson 2012	*	*	*		3	Medium	**	2	Low	*		*	2	Medium
Chew 2012		*	*		2	Medium	*	1	Medium	*	*		2	Medium
Xiang 2011		*	*	*	3	Medium	*	1	Medium	*	*		2	Medium
Kim 2011		*	*	*	3	Medium	*	1	Medium	*		*	2	Medium
Gobl 2011		*	*	*	3	Medium	*	1	Medium	*	*	*	3	Low
Ekelund 2010	*	*	*		3	Medium	*	1	Medium	*	*	*	3	Low
Feig 2013		*		*	2	Medium	**	2	Low	*	*		2	Medium
Ogonowski 2009	*	*	*	*	4	Low	*	1	Medium	*			1	High
Hossein-Nezhad 2009	*	*	*		3	Medium	*	1	Medium	*		*	2	Medium
Russell 2008		*	*	*	3	Medium	**	2	Low	*	*		2	Medium
Rivero 2008		*	*	*	3	Medium	*	1	Medium	*		*	2	Medium
Krishnaveni 2007		*	*	*	3	Medium	*	1	Medium	*	*		2	Medium

Oldfield 2007		*	*	*	3	Medium	*	1	Medium	*	*		2	Medium
Schaefer-Graf 2002	*	*	*		3	Medium	**	2	Low	*			1	High
Zonenberg 2006	*	*	*		3	Medium	*	1	Medium	*	*	*	3	Low
Kousta 2006	*	*	*		3	Medium	*	1	Medium			*	1	High
Jarvela 2006		*	*	*	3	Medium	*	1	Medium		*	*	2	Medium
Cho 2006	*	*	*		3	Medium	**	2	Low	*	*	*	3	Low
Cheung 2006		*	*		2	Medium	*	1	Medium	*	*	*	3	Low
Pallardo 1999		*	*		2	Medium	*	1	Medium	*		*	2	Medium
Buchanan 1999		*	*		2	Medium	*	1	Medium	*		*	2	Medium
Dalfra 2001	*	*	*		3	Medium	*	1	Medium	*	*	*	3	Low
Steinhart 1997		*	*	*	3	Medium	*	1	Medium	*	*		2	Medium
Damm 1992		*	*		2	Medium	*	1	Medium	*	*	*	3	Low
Weijers 2006	*	*	*	*	4	Low	*	1	Medium	*		*	2	Medium
Hunger-Dathe 2006		*	*		2	Medium	*	1	Medium	*	*		2	Medium
Mukerji 2012	*	*		*	3	Medium	*	1	Medium	*	*		2	Medium
Greenberg 1995	*	*	*		3	Medium	*	1	Medium	*			1	High
Lin 2005	*	*	*	*	4	Low	*	1	Medium	*			1	High
Capula 2014		*	*	*	3	Medium	*	1	Medium	*		*	2	Medium
Bentley Lewis 2014		*	*		2	Medium	*	1	Medium	*	*	*	3	Low
Liu 2014		*			1	High	**	2	Low	*			1	High
Lin 2015	*	*	*	*	4	Low	*	1	Medium	*	*		2	Medium
Bao 2015		*	*	*	3	Medium	**	2	Low		*	*	2	Medium

Eades 2015	*	*	*	*	4	Low	*	1	Medium	*	*	*	3	Low
Carvalho Ribeiro 2015	*	*	*		3	Medium	*	1	Medium	*	*	*	3	Low
