# Supplementary material for: Quantification of the type 2 diabetes risk in women with gestational diabetes: a systematic review and meta-analysis of 95,750 women
Source: Diabetologia. 2016 Apr 13;59:1403–11. doi: 10.1007/s00125-016-3927-2 (PMC4901120; doi:10.1007/s00125-016-3927-2)
Supplement: Supplementary file 5 — (PDF 51 kb) [file 125_2016_3927_MOESM5_ESM.pdf]

ESM Search strategy

Search strategy for systematic review on prediction of type 2 diabetes (T2D) in women with gestational diabetes mellitus (GDM)

MEDLINE/EMBASE SEARCH

1. exp Type 2 Diabetes Mellitus/
2. NIDDM.mp.
3. Non-insulin dependent diabetes mellitus.mp.
4. Non-insulin dependent diabetes.mp.
5. type 2 diabetes.mp.
6. type 2 diabetes mellitus.mp.
7. Type II diabetes Mellitus.mp.
8. type II diabetes.mp.
9. Non-insulin-dependent diabetes.mp.
10. Non-insulin-dependent diabetes mellitus.mp.
11. ketosis-resistant diabetes mellitus.mp.
12. Ketosis-resistant diabetes.mp.
13. adult-onset diabetes mellitus.mp.
14. adult-onset diabetes.mp.
15. maturity-onset diabetes mellitus.mp.
16. maturity-onset diabetes.mp.
17. 1 or 2 or 3 or 4 or 5 or 6 or 7 or 8 or 9 or 10 or 11 or 12 or 13 or 14 or 15 or 16
18. gestational diabetes.mp. or exp Diabetes, Gestational/
19. pregnancy induced diabetes mellitus.mp.
20. pregnancy induced diabetes.mp.
21. pregnancy-induced diabetes mellitus.mp.
22. pregnancy-induced diabetes.mp.
23. pregnancy diabetes mellitus.mp.
24. pregnancy diabetes.mp.
25. GDM.mp.
26. Diabetes during pregnancy.mp.
27. gestational diabetes.mp.
28. 18 or 19 or 20 or 21 or 22 or 23 or 24 or 25 or 26 or 27
29. 17 and 28
